# Supplementary material for: The Small RNA Universe of Capitella teleta
Source: Front Mol Biosci. 2022 Feb 25;9:802814. doi: 10.3389/fmolb.2022.802814 (PMC8915122; doi:10.3389/fmolb.2022.802814)
Supplement: Supplementary file 1 [file DataSheet1.ZIP › Supplement/known/CAPTEscaffold_794_29002.pdf]

Provisional ID	:	CAPTEscaffold_794_29002
Score total	:	12216.6
Score for star read(s)	:	3.9
Score for read counts	:	12206.9
Score for mfe	:	1.3
Score for randfold	:	1.6
Score for cons. seed	:	3
Total read count	:	23955
Mature read count	:	21342
Loop read count	:	19
Star read count	:	2594

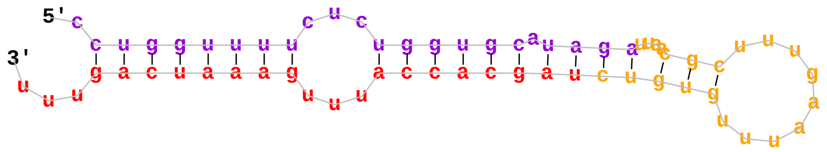

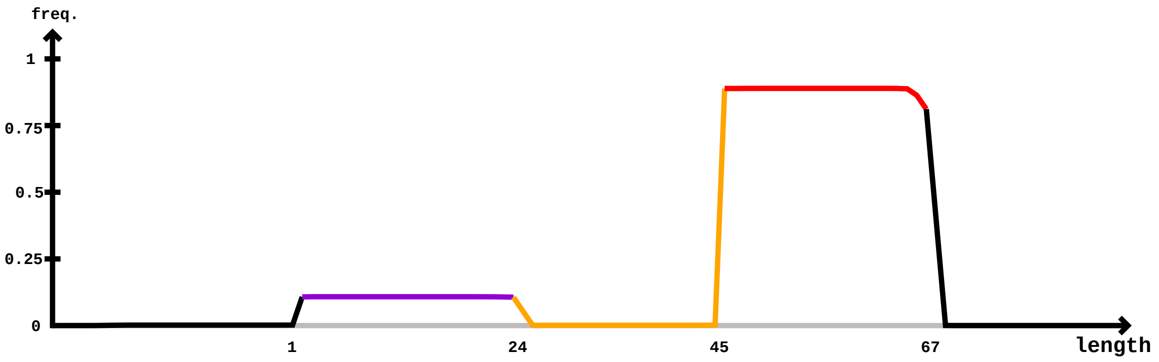

Star **Mature**

```
5'- cgcaugauaauggggcucuggaauccugguuuucucuggugcauagauuaacgcuuugaauuugugucuagcaccauuugaaaucaguuuuccugggugcucacucuccu  -3'   obs
    cgcaugauaauggggcucuggaauccugguuuucucuggugcauagauuaacgcuuugaauuugugucuagcaccauuugaaaucaguuuuccugggugcucacucuccu         exp
    cgcaugauaauggggcucuggaauccugguuuucucuggugcauagauuaacgcuuugaauuugugucuagcaccauuugaaaucaguuuuccugggugcucacucuccu         known
    ..........((.((((.((((..((((((((...((((((.((((....(((.........))))))))))))...))))))))..)))).)))).))........      reads    mm     sample

    ..caugauaauggggcucuggaau......................................................................................      1        0      seq
    ...augauaauggggcucuggaau......................................................................................      7        0      seq
    ....ugauaauggggcucuggaau......................................................................................      13       0      seq
    .....gauaauggggcucuggaau......................................................................................      9        0      seq
    ......auaauggggcucuggaau......................................................................................      11       0      seq
    .......................uccugguuuucucuggugcauag................................................................      3        0      seq
    .......................uccugguuuucucuggugcauaga...............................................................      1        0      seq
    ........................ccugguuuucucuggugca...................................................................      2        0      seq
    ........................ccugguuuucucuggugcau..................................................................      6        0      seq
    ........................ccugguuuucucuggugcaua.................................................................      13       0      seq
    ........................ccugguuuucucuggugcauag................................................................      17       0      seq
    ........................Ucugguuuucucuggugcauaga...............................................................      3        1      seq
    ........................ccugguuuucucuggugcauaga...............................................................      2499     0      seq
    ........................ccugguuAucucuggugcauaga...............................................................      1        1      seq
    ........................ccugguuuucucuggAgcauaga...............................................................      1        1      seq
    ........................ccugguuuucucugguCcauaga...............................................................      1        1      seq
    ........................ccugguuuucucuggugcauagU...............................................................      1        1      seq
    ........................ccuggAuuucucuggugcauaga...............................................................      1        1      seq
    ........................ccuAguuuucucuggugcauaga...............................................................      1        1      seq
    ........................ccugguAuucucuggugcauaga...............................................................      4        1      seq
    ........................ccugguuuucucuAgugcauaga...............................................................      4        1      seq
    ........................ccugguuuucucuggugcauaAa...............................................................      1        1      seq
    ........................ccugguuuucCcuggugcauaga...............................................................      1        1      seq
    ........................ccugguuuucucuggugcaAaga...............................................................      1        1      seq
    ........................ccugguuuucucAggugcauaga...............................................................      2        1      seq
    ........................ccugguuuuAucuggugcauaga...............................................................      1        1      seq
    ........................Ncugguuuucucuggugcauaga...............................................................      1        1      seq
    ........................ccugguuuucucugAugcauaga...............................................................      3        1      seq
    ........................ccugguuuuUucuggugcauaga...............................................................      1        1      seq
    ........................ccAgguuuucucuggugcauaga...............................................................      5        1      seq
    ........................ccugguuuAcucuggugcauaga...............................................................      1        1      seq
```

```
                        Star                                         Mature
cgcaugauaaugggcucuggaauccugguuuucucuggugcauagauuaacgcuuugaauuugugucuagcaccauuugaaaucaguuuuccugggugcucacucuccu
.......................ccuAguuuucucuggugcauagau..............................................................    1      1     seq
.......................ccugguuuucucuggugcauagaAu.............................................................    1      1     seq
........................cugguuuucucuggugcauaga...............................................................    16     0     seq
.........................ugguuuucucuggugcauaga...............................................................    1      0     seq
..............................................uuaacgcuuugaauuugugu...........................................    1      0     seq
..............................................uuaacgcuuugaauuuguguc..........................................    11     0     seq
..............................................uuaacgcuuugaauuugugucu.........................................    7      0     seq
..............................................uuaacgcuuugaauuugugucuagcacc...................................    1      0     seq
.................................................................ucuagcaccauuugaaaucagu......................    4      0     seq
..................................................................cuagcaccauuugaaaucagAu.....................    1      1     seq
..................................................................cuagcaccauuugaaaucaguu.....................    1      0     seq
..................................................................Uuagcaccauuugaaaucaguu.....................    2      1     seq
..................................................................cuagcaccauuugaaaucaguuu....................    13     0     seq
..................................................................Guagcaccauuugaaaucaguuu....................    2      1     seq
..................................................................cuagcaccauuugaaaAcaguuu....................    1      1     seq
..................................................................cuagcaccauuugaaaucaguuuA...................    1      1     seq
...................................................................uagcaccauuugaaauca........................    6      0     seq
...................................................................uagcaccauuugaaaucag.......................    29     0     seq
...................................................................uagUaccauuugaaaucagu......................    1      1     seq
...................................................................uagcaccauuugaaaucagu......................    568    0     seq
...................................................................uagcacUauuugaaaucagu......................    1      1     seq
...................................................................uagcaccauuuAaaaucagu......................    1      1     seq
...................................................................uagcaccauuugaaaucagA......................    3      1     seq
...................................................................Cagcaccauuugaaaucagu......................    1      1     seq
...................................................................Aagcaccauuugaaaucagu......................    1      1     seq
...................................................................uagcaccauuugaGaucaguu.....................    1      1     seq
...................................................................uagcaccauuuAaaaucaguu.....................    2      1     seq
...................................................................uagcaccauuugaUaucaguu.....................    1      1     seq
...................................................................uagcaccauuugaaauAaguu.....................    1      1     seq
...................................................................uagcaccauuugaaUucaguu.....................    1      1     seq
...................................................................uagcaccauuugGaaucaguu.....................    1      1     seq
...................................................................uagcaAcauuugaaaucaguu.....................    1      1     seq
...................................................................Aagcaccauuugaaaucaguu.....................    2      1     seq
...................................................................uagcacAauuugaaaucaguu.....................    2      1     seq
...................................................................uagcaccauuugaaaucaguA.....................    2      1     seq
...................................................................uagcaccaGuugaaaucaguu.....................    1      1     seq
...................................................................uagcaccauuugaaaucaguu.....................    1230   0     seq
...................................................................Cagcaccauuugaaaucaguuu....................    1      1     seq
...................................................................uagcaccauuugaaaucGguuu....................    7      1     seq
...................................................................uagcaccGuugaaaucaguuu....................    4      1     seq
...................................................................uaCcaccauuugaaaucaguuu....................    3      1     seq
...................................................................uagcaccauuAgaaaucaguuu....................    11     1     seq
...................................................................uagcaccauuugaaauAaguuu....................    8      1     seq
...................................................................uaAcaccauuugaaaucaguuu....................    1      1     seq
...................................................................uagAaccauuugaaaucaguuu....................    7      1     seq
...................................................................uUgcaccauuugaaaucaguuu....................    4      1     seq
...................................................................uagcaccauAugaaaucaguuu....................    11     1     seq
...................................................................Gagcaccauuugaaaucaguuu....................    4      1     seq
...................................................................uagcaccauuCgaaaucaguuu....................    4      1     seq
...................................................................uagcaccauuugaaauGaguuu....................    1      1     seq
...................................................................uagcaAcauuugaaaucaguuu....................    7      1     seq
...................................................................uaUcaccauuugaaaucaguuu....................    3      1     seq
...................................................................uagcaccauuugaaaucaguuu....................    18704  0     seq
...................................................................uagcaccauuugaaaAcaguuu....................    9      1     seq
...................................................................uagcaccauuugaaaucaguuA....................    15     1     seq
...................................................................uagcaccauuugaaaucaguGu....................    17     1     seq
...................................................................uagcaccauuugaaauUaguuu....................    5      1     seq
...................................................................uagcCccauuugaaaucaguuu....................    1      1     seq
...................................................................uagUaccauuugaaaucaguuu....................    3      1     seq
...................................................................uagcacUauuugaaaucaguuu....................    12     1     seq
...................................................................uagcaccauuuUaaaucaguuu....................    1      1     seq
...................................................................uagcaccauuugaaaucagGuu....................    3      1     seq
...................................................................uagcaccauuugaaaucaguuG....................    3      1     seq
...................................................................uagcacGauuugaaaucaguuu....................    4      1     seq
...................................................................uagcaccauuugaaaGcaguuu....................    1      1     seq
...................................................................uagcaccauuugaaaCcaguuu....................    2      1     seq
...................................................................uagcaccauuuAaaaucaguuu....................    61     1     seq
...................................................................uagcaccaAuugaaaucaguuu....................    2      1     seq
...................................................................uagcaccauuugaGaucaguuu....................    4      1     seq
...................................................................uagcaccaGuugaaaucaguuu....................    1      1     seq
```

```
                        Star                                        Mature
cgcaugauaauggggcucuggaauccugguuuucucuggugcauagauuaacgcuuugaauuugugucuagcaccauuugaaaucaguuuuccugggugcucacucuccu
....................................................................uagcaccauuugUaaucaguuu....................   2    1    seq
....................................................................uagcaUcauuugaaaucaguuu....................   3    1    seq
....................................................................Aagcaccauuugaaaucaguuu....................   73   1    seq
....................................................................uagcaccauCugaaaucaguuu....................   2    1    seq
....................................................................uagcaccauuugaaaucaguuC....................   4    1    seq
....................................................................uNgcaccauuugaaaucaguuu....................   1    1    seq
....................................................................uagcaccauuugaaGucaguuu....................   7    1    seq
....................................................................uagcaccauuugaaUucaguuu....................   3    1    seq
....................................................................uCgcaccauuugaaaucaguuu....................   1    1    seq
....................................................................uagcaccauuugaaaucagAuu....................   6    1    seq
....................................................................uagcaGcauuugaaaucaguuu....................   1    1    seq
....................................................................uagcGccauuugaaaucaguuu....................   4    1    seq
....................................................................uagcUccauuugaaaucaguuu....................   5    1    seq
....................................................................uagcaccauuuCaaaucaguuu....................   1    1    seq
....................................................................Nagcaccauuugaaaucaguuu....................   6    1    seq
....................................................................uagcaccauuugaaaucaguAu....................   7    1    seq
....................................................................uagcacAauuugaaaucaguuu....................   32   1    seq
....................................................................uagcaccauuugaUaucaguuu....................   1    1    seq
....................................................................uagcaccauuugGaaucaguuu....................   7    1    seq
....................................................................uagcaccauuugaaaucaguuuA...................   194  1    seq
....................................................................uagcaccauuAgaaaucaguuuu...................   1    1    seq
....................................................................uagcaccauuugaaaucaguuuu...................   158  0    seq
....................................................................uagcaccauuugaaaucaguuuuA..................   4    1    seq
....................................................................uagcaccauuugaaaucaguuuuU..................   2    1    seq
.....................................................................agcaccauuugaaaucaguu.....................   1    0    seq
......................................................................gcaccauuuUaaaucaguuu....................   1    1    seq
......................................................................gcaccauuugaaaucaguuu....................   4    0    seq
.......................................................................caccauuugaaaucaguuu....................   3    0    seq
........................................................................accauuugaaaucaguuu....................   2    0    seq
.........................................................................ccauuugaaaucaguuuA...................   1    1    seq
```
